# The Spatial-Temporal Characteristics of Air Pollution in China from 2001–2014

**DOI:** 10.3390/ijerph121215029

**Published:** 2015-12-15

**Authors:** Junzhe Bao, Xiping Yang, Zhiyuan Zhao, Zhenkun Wang, Chuanhua Yu, Xudong Li

**Affiliations:** 1 Department of Epidemiology and Biostatistics, School of Public Health, Wuhan University, # 185 Donghu Road, Wuhan 430071, China; junzhe_bao@126.com (J.B.); wongzhenkun@gmail.com (Z.W.); 2 State Key Laboratory of Information Engineering in Surveying, Mapping and Remote Sensing, Wuhan University, 129 Luoyu Road, Wuhan 430079, China; 0yangxiping0@163.com (X.Y.); zhaozhiyuan@whu.edu.cn (Z.Z.); 3 Global Health Institute, Wuhan University, # 8 Donghunan Road, Wuhan 430072, China; 4 Office of Epidemiology, Chinese Center for Disease Control and Prevention, 155 Changbai Road, Changping District, Beijing 102206, China

**Keywords:** Air Pollution Index, Air Quality Index, spatial-temporal characteristics, shift of gravity center

## Abstract

To provide some useful information about the control of air pollution in China, we studied the spatial-temporal characteristics of air pollution in China from 2001–2014. First, we drew several line charts and histograms of the Air Pollution Index (API) and Air Quality Index (AQI) of 31 capital cities and municipalities to research the distribution across different times and cities; then, we researched the spatial clustering of API and AQI; finally, we examined the shift of the gravity center of API and AQI in different years and months. The API values had a decreasing trend: the high values had a clustering trend in some northern cities, and the low values had a clustering trend in some southern cities. The AQI values were relatively low, from 15:00–17:00 during the day. The gravity center of API had a trend of moving south from 2001–2003, then fluctuated in an unordered pattern and moved north in the winter. The AQI gravity center did not have a regular shift during different months. In conclusion, the government should take action to mitigate air pollution in some typical cities, as well as air pollution during the winter.

## 1. Introduction

Over the past ten years, due to rapid industrialization and economic development, China has experienced deteriorating air quality. A study undertaken by the World Bank in 2008 reported that China had 16 of the 20 most air-polluted cities in the world; furthermore, according to the State Environmental Protection Agency (SEPA), two-thirds of the urban population breathed air of substandard quality [1]. The Air Pollution Index (API) was used to evaluate the quality of air in China from 2001–2012. The index synthesizes SO_2_, NO_2_ and PM_10_ information into a comprehensive value, thus making it convenient for people to understand the condition of air pollution. However, people have typically found discrepancies between the API values and their perceptions of air pollution. That is to say, they often feel uncomfortable during the day, although the API values are not as high during the day, meaning that the API may not reflect air quality accurately. Thus, the Air Quality Index (AQI) was adopted to replace the API in China. This index synthesizes information about SO_2_, NO_2_, PM_10_, PM_2.5_, CO and O_3_. Additionally, the hourly AQI values and concentrations of single pollutants are reported on the web to provide health guidance to the public [2].

In China, air pollution may display different characteristics in different districts and periods. Many previous studies on air pollution in China have focused on certain areas or were limited to relatively short periods [3,4,5,6]. Spatial-temporal analyses of the AQI on a national scale in China are relatively scarce thus far. Wang *et al.* studied the spatial and temporal variations of six criteria that assess air pollutants, which were used to calculate the AQI in 31 capital cities and municipalities in China from March 2013–February 2014. Their study period included 12 months, but not a complete calendar year [7]. Furthermore, studies comparing the API and AQI and the shift of the API and AQI gravity centers on a national scale are relatively few in China. 

In this study, we aimed to research the spatial-temporal characteristics of air pollution in China from 2001–2014. We studied the changing trend of API in 31 capital cities and municipalities in mainland China from 2001–2012, their distribution in different years and months, their spatial cluster and the shift of their gravity centers in different years and months. Additionally, we studied these aspects of AQI in 2014, as well as the distribution of AQI at different times of day. We intend to provide some useful macro-level information and to contribute to the prevention and control of air pollution in China.

## 2. Material and Methods 

### 2.1. Site Information

**Figure 1:**
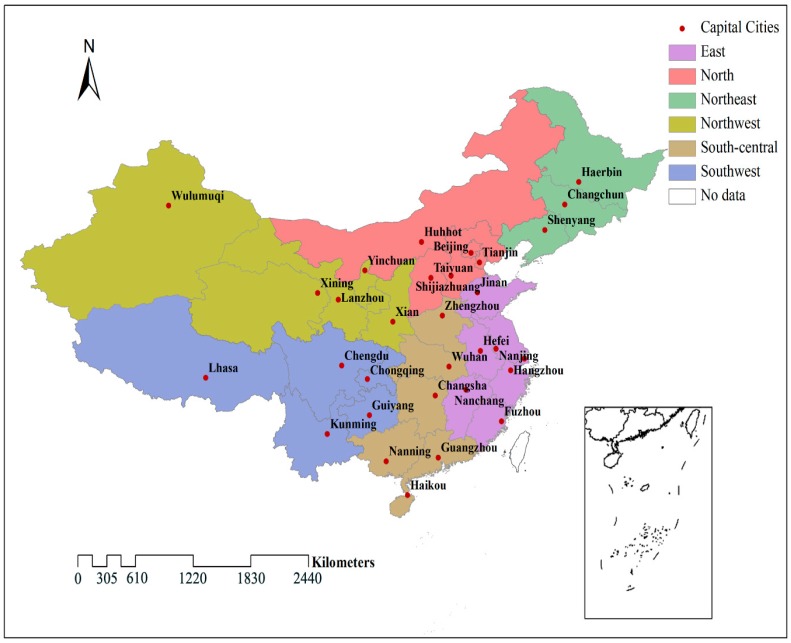
The distribution of the researched cities.

Twenty-seven capital cities and 4 municipalities in mainland China were chosen as pilot cities. The distribution of the 31 cities is shown in Figure 1, and the description of them is shown in Appendix Table A1.

### 2.2. Data Sources

API data from January 2001–December 2012 were acquired from the Ministry of Environmental Protection of China website [8] using the XML package in R, whereas the AQI data from January–December of 2014 were acquired using the HTMLParser package in the C # language. The API data contained the daily API value, which was calculated after the day it represented, and its spatial scale was at the city level; whereas the AQI data contained the hourly AQI values and the hourly concentrations of PM_2.5_, PM_10_, O_3_, NO_2_, SO_2_ and CO, and its spatial scale was at the district level in the city. As for AQI, we adopted the average AQI value of different districts of a city to reflect the air quality of that city. In this study, we analyzed the corresponding data of 31 capital cities and municipalities in mainland China.

The API was calculated as follows:

(1) $${\text{API}}_{i}=\frac{API_{U}-API_{L}}{C_{U}-C_{L}}\left(C_{i}-C_{L}\right)+API_{L}$$

(2) $$\text{API}=\text{max}\left(API_{i}\right)$$

where API*_i_* is the index for pollutant *i* (*i.e.*, PM_10_, SO_2_ and NO_2_); C*_i_* is the daily average concentration of pollutant *i*; C*_U_* and C*_L_* are the upper and lower breakpoints corresponding to C*_i_*, respectively; and API*_U_* and API*_L_* are the breakpoints of API corresponding to C*_U_* and C*_L_*, respectively. The overall API is the maximum API*_i_* (Table 1) [9].

**Table 1:** The concentration breakpoints for each pollutant in the calculation of the Air Pollution Index (API).

Pollutant Concentrations(μg/m^3^)			API
PM_10_	SO_2_	NO_2_	
0	0	0	0
150	150	120	100
350	800	280	200
420	1600	565	300
500	2100	750	400
600	2620	940	500

The AQI was calculated as follows:

(3) $$AQI_{i}=\frac{IAQI_{Hi}-IAQI_{Lo}}{C_{Hi}-C_{Lo}}\left(C_{i}-C_{Lo}\right)+IAQI_{Lo}$$

(4) $$AQI=max\left(AQI_{i}\right)$$

where AQI*_i_* is the index for the pollutant *i* (*i.e.*, SO_2_, NO_2_, CO, O_3_, PM_2.5_ and PM_10_); C*_i_* is the monitored ambient concentration of pollutant *i*; C*_Hi_* and C*_Lo_* are the nearly upper and lower breakpoints corresponding to C*_i_*, respectively; IAQI is the individual air quality index, and IAQI*_Hi_* and IAQI*_Lo_* are the sub-indices corresponding to C*_Hi_* and C*_Lo_*, respectively. The overall AQI is the maximum sub-AQI of all pollutants (Table 2) [10].

**Table 2:** The concentration breakpoints for each pollutant in the Air Quality Index (AQI) calculation.

IAQI	Pollutant Concentrations (μg/m^3^)					
	SO_2_	NO_2_	CO	O_3_	PM_2.5_	PM_10_
0	0	0	0	0	0	0
50	150	100	5	160	35	50
100	500	200	10	200	75	150
150	650	700	35	300	115	250
200	800	1200	60	400	150	350
300	1600	2340	90	800	250	420
400	2100	3090	120	1000	350	500
500	2620	3840	150	1200	500	600

### 2.3. Methodology

First, we drew the API line charts to analyze the changing trend of the API from 2001–2012. For the presentation of the results, we divided the 31 cities into 6 regions (the northeast, north, northwest, east, south-central and southwest of China), which derived from the 6 administrative regions set up after the establishment of the People’s Republic of China; then, we drew the API and AQI histograms in different months and different cities to study their distribution. Additionally, we drew AQI histograms at different times so that we could preliminarily know the times at which the AQI was relatively high or low.

We then studied the spatial clustering of the API and AQI. We took the global spatial autocorrelation using the univariate Moran’s I method; the spatial weight was set based on the spatial proximity of corresponding provinces, and the randomization was set as “999 permutations”. We further researched the local spatial autocorrelation using the univariate local Moran’s I method. In the weight setting section, we chose Queen contiguity, and the order of contiguity was set as “1”. Because Hainan is an island, we artificially set Haikou, Guangzhou and Nanning as neighbors. In the latter method, the “high-high” clusters indicate that the marked areas (usually marked by bright red) possess relatively high values of API or AQI and that they are surrounded by areas with high values, which usually indicates that these areas need further research and special attention to reduce the pollution. “Low-low” clusters indicate that the marked areas (usually marked by deep blue) possess relatively low values and are surrounded by areas with low values. “High-low” clusters indicate that the marked areas (usually marked by light red) possess relatively high values, but are surrounded by areas with low values. “Low-high” clusters indicate that the marked areas (usually marked by light blue) possess relatively low values, but are surrounded by areas with high values. 

Finally, we studied the shift of the API gravity center from 2001–2012 and from January–December, as well as the shift of the AQI gravity center from January–December. The point corresponding to the center of gravity was calculated as follows:

(5) $$\bar{\text{x}}=\frac{\sum_{i=1}^{n}x_{i}API_{i}}{\sum_{i=1}^{n}API_{i}}\;\bar{\text{y}}=\frac{\sum_{i=1}^{n}y_{i}API_{i}}{\sum_{i=1}^{n}API_{i}}$$

where $\bar{\text{x}}$ and $\bar{\text{y}}$ represent the longitude and latitude of the center of gravity, respectively; x*_i_* and y*_i_* represent the longitude and latitude of a certain city, respectively; and *API_i_* represents the corresponding API value of that city. 

The line charts, histograms and the shift of the gravity center were drawn by R 3.2.0, and the spatial clustering was analyzed and drawn by GeoDA 1.6.7.

## 3. Results 

### 3.1. The Changing Trend of API from 2001–2012

**Figure 2:**
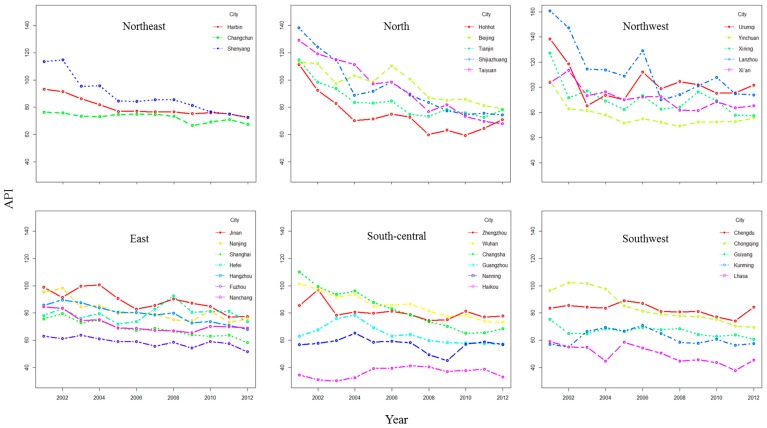
The changing API trends from 2001–2012.

From Figure 2, we can see that the API values of most of the northern cities had a decreasing trend from 2001–2012 and that they had a relatively sharp decrease from 2001–2004. The API values of most of the southern cities had relatively small fluctuations; the API values of northern cities were generally higher than those of southern cities, with the northwest region having the relatively highest API values. The API values of some cities had an increasing trend in 2012, compared to 2011, such as Hohhot, Urumqi, Chengdu and Lhasa.

### 3.2. The Distribution of API and AQI in Different Cities, Months and Hours

**Figure 3:**
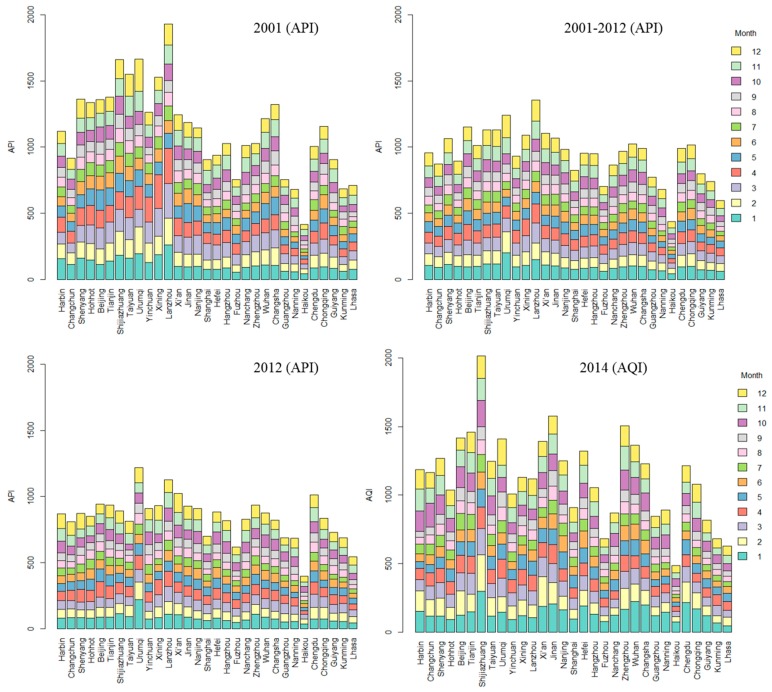
The distribution of API and AQI in different cities and months.

**Figure 4:**
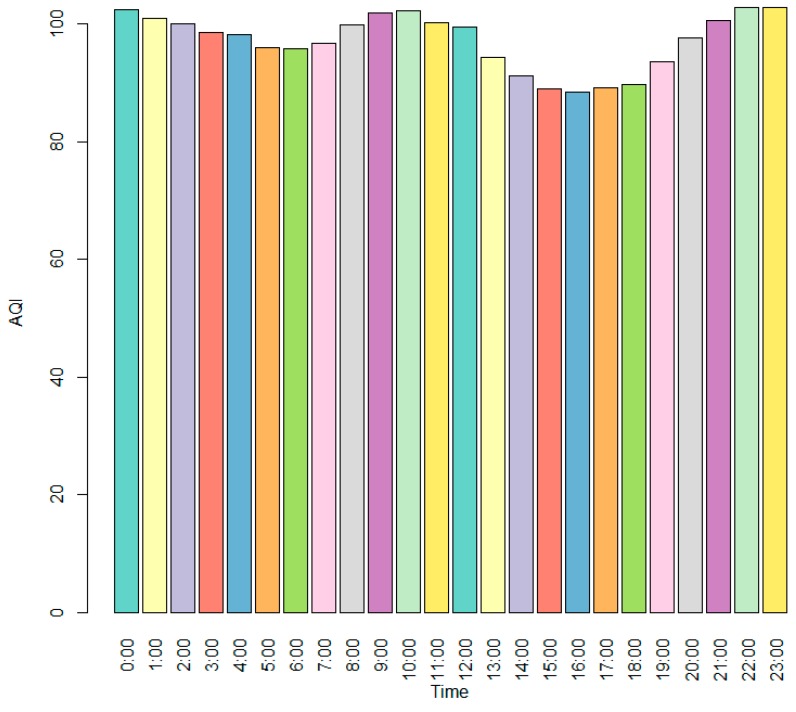
The distribution of the AQI at different hours.

Figure 3 shows that from 2001–2012, the cities of Lanzhou, Urumqi and Beijing had the highest API values; and the cities of Shijiazhuang, Jinan and Zhengzhou had the highest AQI values in 2014. We also found that the cumulative API values were higher in northern cities than in southern cities, but the gap became narrow with time. The API and AQI values had a different distribution in different months. Generally, they had relatively high values in November, December, January and February, which are the winter months in China; and relatively low values in June, July, August and September, which are the summer months. The AQI values were relatively low from 15:00–17:00, which is to say that they were relatively low during the afternoon (Figure 4).

### 3.3. The API and AQI Spatial Clustering 

**Figure 5:**
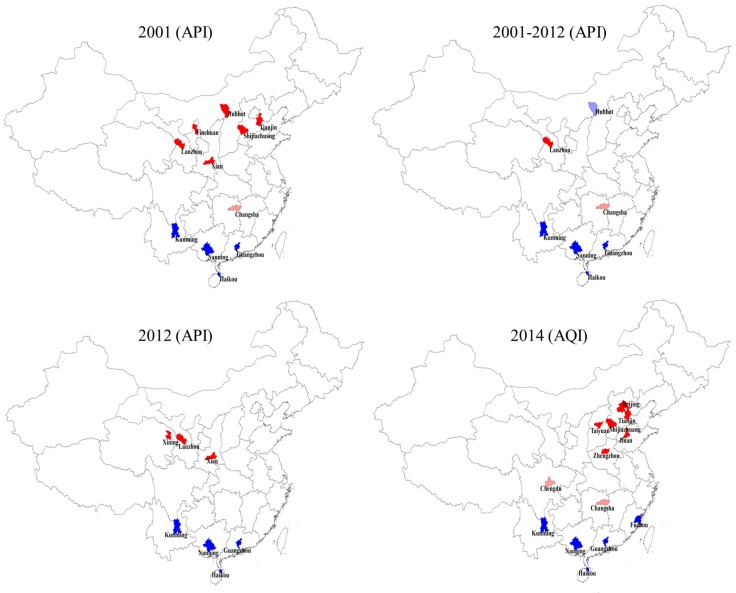
The local spatial autocorrelation of the API and AQI. The bright red represents “high-high” clusters; the deep blue represents “low-low” clusters; the light red represents “high-low” clusters; and the light blue represents “low-high” clusters.

From Table 3, we can see that the API and AQI values had a spatial cluster trend, and Figure 5 further displays the cluster characteristics. As for the API, its values had a high-high cluster (the cities with bright red) in northern cities, especially in northwestern cities, such as Lanzhou, and had a low-low cluster in southern cities (the cities with deep blue), such as Kunming, Nanning, Guangzhou and Haikou. Hohhot’s API was relatively low (shown with light blue) compared to those of its surrounding cities, whereas Changsha’s API was relatively high (shown with light red) compared to those of its surrounding cities. As for the AQI, its values had a high-high cluster in Shijiazhuang, Beijing, Tianjin, Jinan, Taiyuan and Zhengzhou and a low-low cluster in Kunming, Nanning, Guangzhou and Haikou. Changsha and Chengdu’s AQI values were relatively high compared to those of their surrounding cities.

**Table 3:** The global spatial autocorrelation of the API and AQI.

Indicators	*Moran’s I*	*Z*	*p*
API (2001)	0.4711	4.2833	0.001
API (2001–2012)	0.3682	3.4336	0.001
API (2012)	0.3028	2.9888	0.006
AQI (2014)	0.4909	4.6800	0.001

### 3.4. The Shift of the API and AQI Gravity Center 

**Figure 6:**
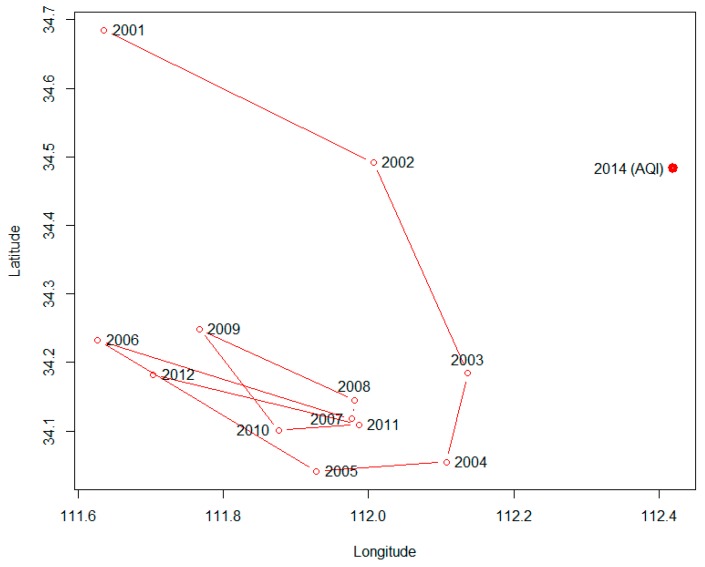
The shift of the API gravity center from 2001–2012.

**Figure 7:**
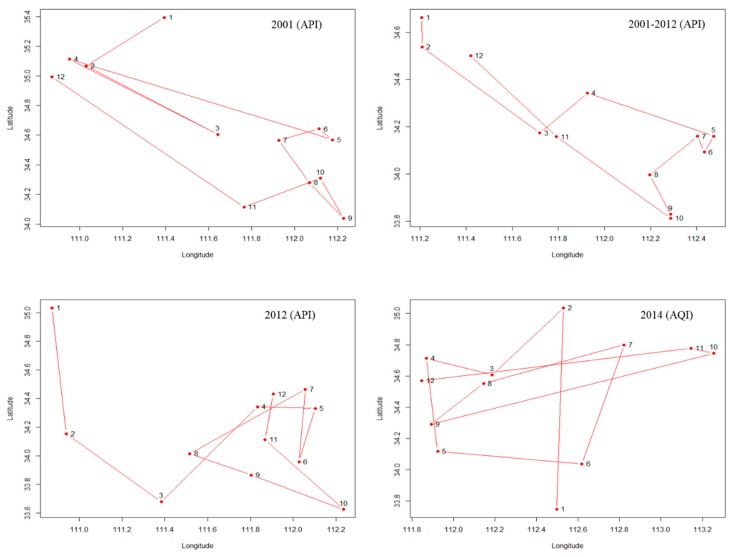
The shifts of the API and AQI gravity centers in different months.

Figure 6 shows that the API gravity center shifted from north to south slightly from 2001–2003 and then fluctuated slightly without order. The AQI gravity center deviated to the east compared to the API. As for the API, its gravity center shifted to the north in December, January and February and to the south in August, September and October. As for the AQI, its gravity center did not have an obvious trend in different months (Figure 7).

## 4. Discussion

We found that the API values had a declining tendency from 2001–2012 and that they were higher in the northern cities than in the southern cities, which is consistent with the findings of some previous studies performed in China [11,12,13]. These previous studies found that PM_10_ was the primary pollutant throughout the year, and the source appointment of PM_10_ indicated that PM_10_ was mainly generated from re-suspended dust and coal combustion [14,15,16]. The decline of the API may be caused by a variety of factors, such as the 10th and 11th Five-Year Plan established from 2001–2010 in China and, specifically the strengthened particulate matter emission standard for power plants issued in 2003 (GB 13223-2003) [17], the efficient and clean use of coal and the replacement of oil and coal with natural gas and moving the polluting enterprises to the suburbs. The northern cities consumed more energy for warmth in the cold season, and there were more polluting firms in the northern cities than in the southern cities. Meteorological and geographical factors also contributed to the phenomenon of higher API values in the northern cities; for example, there were more sandstorms and less rainfall in the northern cities [6].

The API values were higher in cold months than hot months, which may be because there was less rainfall in winter, the climate was dry and the vegetation withered and flying dust and sandstorms occurred with the addition of the northwest wind. Additionally, because of cold temperatures, inversion layers form easily during the winter and hinder the diffusion of pollutants. The consumption of energy for warmth worsens this situation. In contrast, there was more rainfall in the summer; the climate was relatively moist and the vegetation cover increased; and the solar radiation was strong and resulted in increased ground temperatures. Thus, the inversion was not easily formed, and the air convection increased, which promotes the spread of pollutants [18]. The AQI values were relatively low during the afternoon, possibly because the temperature in the afternoon is relatively high and air convection is frequent.

The API values were high in Lanzhou and Urumqi, and the high API values had a clustering trend around Lanzhou. The reasons for this phenomenon may include the dry climate in northwestern China and the relatively sparse vegetation and rainfall, which facilitated the formation of sand-dust weather. Additionally, the weather was cold in the winter, and more coal was consumed for heating in this region. As for Lanzhou, in addition to the above-mentioned factors, it is a heavy industry city, with serious industrial pollution. Furthermore, Lanzhou is located in the river valley of the Loess Plateau and belongs to the basin terrain, which prevents the diffusion of pollutants.

The AQI values were high in Shijiazhuang, Jinan and Zhengzhou in 2014, and the high values had clustering trends around these cities. This distribution and clustering trend of AQI had some fine differences compared to the API. The possible reasons for this phenomenon may include: the different study period; and that the AQI reflects more pollutant information compared to the API, such as PM_2.5_, O_3_ and CO. Some of these pollutants are primarily generated from industrial pollution, vehicle exhaust and incineration [19,20], instead of flying dust, which is more common in the northwest of China and was a main source of PM_10_.

The gravity center of API had a trend of moving south from 2001–2003. This phenomenon demonstrated that the pollution problems had been mitigated in northern China. The API gravity center had a trend of moving to north in the months of December, January and February, and this phenomenon demonstrated that the pollution problems became more serious during the winter in northern China and might be caused by coal heating and climate conditions. As for the AQI, the gravity center did not have obvious changing characteristics from January–December. This lack of change might be caused by the AQI, including other constituents, such as PM_2.5_, O_3_ and CO, which are not included in the API, and these indicators varied less between the north and south of China, or the difference may have been offset by the constituents of API. Additionally, the API and AQI data were from different years, as mentioned above, and an increasing number of northern cities have been using natural gas or other clean energy for heating and cooking, rather than coal, in recent years, which helps reduce the difference in pollution in the winter between the north and the south. The AQI data included only one year, so this phenomenon requires further research.

This study has some limitations. First, the API or AQI data from 2013 were not included, because most of the studied cities started to replace the API with AQI in 2013, and the published AQI data had some deficiencies in 2013. Second, we mainly researched the spatial-temporal characteristics of the API and AQI and did not examine specific pollutants. In the future, we will further research the spatial-temporal characteristics of some specific pollutants, as well as their possible influencing factors and the health impact of air pollution. 

## 5. Conclusions 

The air pollution of northern cities is relatively more serious than that of southern cities in China, but the gap has been narrowing with time. Air pollution is relatively mild during the afternoon. Air pollution has a clustering trend, with high pollution areas clustered in the north and low pollution areas clustered in the south. The air pollution is more serious in the winter and is relatively milder in the summer. 

## Appendix

**Table A1:** Description of the 31 cities examined in this study *.

City	Province (Autonomous Region)	Urban Area (km^2^)	Precipitation (mm)	Average Air Temperature (°C)	Average Relatively Humidity (%)	Population (Million)	Gross Industrial Output Value (Billion RMB)	Freight Traffic (Megatons)	Passenger Traffic (Million)	Industrial Soot and Dust Emission (Tons)	SO_2_ Emission (Tons)
Harbin	Heilongjiang	53,068	664.1	3.6	70.4	9.94	285.16	117.64	156.18	52,257	80,740
Changchun	Jilin	20,571	717.6	4.6	63.1	7.57	829.42	162.27	144.47	40,803	69,046
Shenyang	Liaoning	12,860	678.1	6.4	71.2	7.25	1270.23	217.19	328.69	53,191	96,756
Hohhot	Inner Mongolia	17,224	562.6	7.1	46.9	2.3	130.49	147.33	29.27	18,372	99,375
Beijing	Municipality	16,410	733.2	12.9	50.9	12.97	1559.62	262.91	1490.37	30,844	59,330
Tianjin	Municipality	11,946	614.5	12.6	57.2	9.93	2342.75	464.75	284.62	59,036	215,481
Shijiazhuang	Hebei	15,848	650.5	13.9	54.6	10.05	764.31	287.55	153.78	98,364	179,942
Taiyuan	Shanxi	6988	448.8	10.7	51.2	3.66	258.87	142.25	53.57	42,084	101,780
Urumqi	Xinjiang Uygur	14,216	286.3	7.4	52.8	2.58	215.08	182.18	49.1	51,109	109,802
Yinchuan	Ningxia Hui	9461	182.4	9.8	48.1	1.67	164.76	135.2	37.34	26,348	105,743
Xining	Qinghai	7649	448.6	5.2	59	1.98	103.12	32.78	52.33	49,367	71,408
Lanzhou	Gansu	13,085	255.5	8.3	53	3.22	208.44	97.28	48.31	33,598	70,151
Xi'an	Shaanxi	9983	423.9	15.8	58	7.96	402.32	449.24	361.54	17,463	83,063
Jinan	Shandong	8177	578.4	14.4	55.2	6.09	424.83	262.08	174.88	51,609	103,187
Nanjing	Jiangsu	6597	799.8	16	68.1	6.38	1143.78	389.41	469.92	40,679	119,155
Shanghai	Municipality	6340	1004.9	16.9	69.5	14.27	3189.69	941.9	174.02	87,100	240,100
Hefei	Anhui	11,408	819	16.5	73.1	7.11	660.01	337.2	344.18	41,120	45,572
Hangzhou	Zhejiang	16,596	1617.2	17.2	70.7	7.01	1296.23	300.88	358.19	33,015	86,181
Fuzhou	Fujian	11,968	1818.7	20.2	75.2	6.55	595.49	172.13	192.79	37,487	76,225
Nanchang	Jiangxi	7402	1793.9	18	77	5.08	385.65	95.25	106.24	11,115	43,470
Zhengzhou	Henan	7446	501	15.5	53.2	10.73	941.30	266	356.6	51,242	141,246
Wuhan	Hubei	8494	1162.6	16.4	81.4	8.22	1006.57	438.92	274.93	20,496	100,072
Changsha	Hunan	11,819	1731	17.6	76.3	6.61	705.83	259.7	364.41	11,976	21,209
Guangzhou	Guangdong	7434	1816.4	21.7	81.5	8.22	1606.64	751.75	760.69	12,600	66,600
Nanning	Guangxi Zhuang	22,112	870.8	21.4	79.6	7.14	210.93	297.85	120.42	25,012	30,626
Haikou	Hainan	2304	1740.4	24.6	81.7	1.62	51.60	104.13	401.16	727	1834
Chengdu	Sichuan	12,390	1343.3	16.9	77.1	11.73	784.91	395.42	1068.74	24,723	56,730
Chongqing	Municipality	82,402	1105.1	18.3	71.7	33.43	1309.51	1101.36	1577.98	166,142	509,788
Guiyang	Guizhou	8083	1260.4	13.7	84.5	3.75	159.29	166.35	464.9	19,743	65,259
Kunming	Yunnan	21,473	814.1	16.3	66.8	5.43	301.13	261.93	159.18	58,332	113,277
Lhasa	Tibet	29,518	367	9.6	33.5	0.56	5.83	4.77	8.6	647	1075

*The data were derived from China Statistical Yearbook 2013.
